# The Perception of Vitamins and Their Prevalence in Fortified Food and Supplements in Japan

**DOI:** 10.3390/nu13093136

**Published:** 2021-09-08

**Authors:** Tsuyoshi Chiba, Nanae Tanemura, Chiharu Nishijima

**Affiliations:** Department of Food Function and Labeling, National Institute of Health and Nutrition, National Institutes of Biomedical Innovation, Health and Nutrition, 1-23-1 Toyama, Shinjuku-ku, Tokyo 162-8636, Japan; n-tanemura@nibiohn.go.jp (N.T.); c-nishijima@nibiohn.go.jp (C.N.)

**Keywords:** vitamins, fortified food, dietary supplements, nutritional status

## Abstract

Most vitamins are primarily ingested from foods. However, it has been reported that intakes of some vitamins do not meet the nutrition reference values even in high-income countries. In this case, vitamin-fortified foods and/or vitamin supplements are helpful to fix insufficient/deficient status. However, it is not clear whether consumers are aware of their nutritional status or whether they use these products efficiently. To address this issue, we conducted an online cross-sectional questionnaire survey among 14,741 Japanese adults (over 20 years old, 7489 males and 7252 females) concerning the perceptions and prevalence of vitamin-fortified food and/or vitamin supplements. Differences in distribution among groups were compared using the chi-squared test. According to dietary habits, 33.2% of the participants consumed a well-balanced diet every day, but 25.5% could not because of time (41.6%) or money (36.9%) constraints. The perception of each vitamin varied: the highest was for vitamin C (93.2%) and the lowest was for biotin (41.9%). In addition, only a portion of the participants believed that they took sufficient amounts of each vitamin; the highest was vitamin C (22.3%) and the lowest was biotin (5.2%). Despite this situation, most did not use vitamin-fortified food and/or vitamin supplements due to economic reasons. Among vitamin-fortified food and/or vitamin supplement users, the purposes for the usage of these products were varied, such as maintaining health (80.5%), supplementation of nutrients (47.8%), beauty-related purposes (27.5%), and to prevent infectious disease (23.2%). To remedy nutritional status in individuals, it is important to improve not only consumer awareness but also the environment, which can lead consumers to use acceptable vitamin products without any burden.

## 1. Introduction

Vitamins are essential micronutrients and are important for the maintenance of health in everyone. Dietary reference intakes (DRIs) are a general term for a set of reference values used to plan and assess the nutrient and energy intakes of healthy people, which vary by age and sex [1]. DRIs for Japanese people (DRIs-J) have a revision every five years, and the latest revision was made in 2020 [2]. The DRIs-J include estimated average requirement (EAR), recommended dietary allowance (RDA), adequate intake (AI), tolerable upper intake level (UL), and tentative dietary goals for preventing lifestyle-related diseases (DG). However, the DRIs-J are reference values of nutrition intakes for Japanese people, meaning that they are not the actual intakes of nutrition among Japanese people. To assess the actual intakes of nutrition among Japanese people, the National Health and Nutrition Survey Japan (NHNS-J) is conducted every year, except in 2020 because of the novel coronavirus disease 2019 (COVID-19). Comparing DRIs-J and NHNS-J, it is clear that the intake levels of some vitamins and minerals are below RDA (EAR, RDA, or AI). It is reported that accurate measurement of nutrients from dietary supplements is critical in precision nutrition [3]. In this situation, consumers should care for their nutrition intake not only from dairy foods but also from nutrient-fortified foods and/or supplements.

In spite of the detailed evidence regarding nutrition, double-burden malnutrition is a major issue in most low-income and middle-income countries [4]. Malnutrition is also a concerning issue in high-income countries [5,6,7]. The National Health and Nutrition Examination Survey 2007–2010 in the USA showed the that vitamins A, C, D, E, K, folate, magnesium, calcium, potassium, fiber, and long chain omega-3 polyunsaturated fatty acids are under-consumed compared to the EAR in almost all population groups [8]. Among these nutrients, there is a prevalence of over 6% of vitamin B_6_, B_12_, C, and D deficiency in several sub-populations. In Japan, there are few studies on malnutrition. One study showed that skipping breakfast is associated with deficiencies of many vitamins and minerals such as vitamins A, B_1_, and B_2_, and some minerals among Japanese female junior high school students [9]. Another study showed that people who frequently ate meals prepared away from home had a lower intake of vitamin C and some minerals [10]. These results showed the possibility of malnutrition in some populations in Japan, so exhaustive research is needed.

Food labeling is the most powerful tool to understand the amount of nutrients we can consume from each food. In 2020, the “food labeling act” came into force in Japan, after 5 years of the enforcement grace period. Under the “food labeling act”, the labeling of energy, protein, fat, carbohydrates, and salt equivalent on product packages is mandatory. On the other hand, the labeling of saturated fat and dietary fiber is recommended, and for other ingredients, including vitamins, minerals and fatty acids, labeling is voluntary. This means that we can check energy, protein, fat, carbohydrates, and salt intake with the labeling but not for other nutrients including vitamins. However, vitamin/mineral contents, which include those both originating from foods and fortified, are labeled on vitamin-/mineral-fortified foods. In recent decades, the prevalence of dietary supplement use, especially vitamins and minerals, has increased not only in elderly persons but also in younger persons, including adolescents [11,12,13]. In addition, mothers also give their children dietary supplements [14]. Today, there are many ingredients, such as herbs, which claim diverse purposes. However, dietary supplements of vitamins and minerals are still the most popular in all generations for the maintenance of health, and these might complement insufficiency/deficiency of nutrients [11,12,13,14]. In addition, many vitamin/mineral-fortified foods are also marketed in Japan, and people can buy these products at supermarkets, drug stores or online markets. Besides regular foods (milk, yogurt, cereal, etc.), vitamin-/mineral-fortified foods can be incorporated in the diet.

In Japan, many people may be familiar with some vitamins, such as vitamin B, vitamin C, and vitamin E, but not others, such as biotin and niacin [11,12,13,14]. In this study, we conducted an online cross-sectional questionnaire survey to clarify the knowledge and prevalence of each vitamin of consumers and whether consumers use vitamin-fortified food/vitamin supplements and whether they are aware of the level of their intake of each vitamin.

## 2. Materials and Methods

### 2.1. Participants and Procedures

An online cross-sectional questionnaire survey was conducted by Cross Marketing Inc. (Tokyo, Japan) from 2 to 4 February 2021. The 14,741 respondents comprised 7489 (50.8%) males and 7252 (49.2%) females. This study was conducted with the approval of the Research Ethics Committee of the National Institutes of Biomedical Innovation, Health and Nutrition (No. 13-6, approved on 26 January 2021), and in accordance with the Declaration of Helsinki. The question items were presented to the research company, and the research company then conducted an Internet survey, collected the survey results, and anonymized personal information to deliver data that could not be identified by an individual. The participants were aged 20 and over and were registered as survey monitors with the research company. An email with a survey cooperation request and a webpage link to the survey was sent to computer-randomized monitors. An explanation of this study was provided at the beginning of the survey page, and only those individuals who had agreed to participate answered the questionnaire. Complete responses were collected on a first come, first served basis, until the numbers reached the quotas for sex and age. Questions were asked about the usual dietary status, vitamin-fortified food or/and vitamin supplement usage status, awareness of each vitamin, and intake status (Supplementary File). This was the first survey about perceptions of each vitamin and their prevalence in fortified food and supplements. Therefore, the questionnaire was not previously validated by others. However, we conducted the internet questionnaire about dietary supplement use several times, and the prevalence of dietary supplements were almost same among other surveys [15,16] and compared to a paper survey [11].

### 2.2. Statistical Analysis

Differences in distribution among groups (frequency of a well-balanced diet, vitamins, sex) were compared using the chi-squared (χ^2^) test. All statistical analyses were performed using Cross finder 2 ver.2.3.2.0 provided by the internet survey company (Cross Marketing Inc., Tokyo, Japan), and a *p*-value of <0.05 was considered statistically significant.

## 3. Results

### 3.1. Characteristics

The 14,741 respondents (45.8 ± 15.2 years old) comprised 7489 males (46.4 ± 15.3 years old) and 7252 females (45.2 ± 15.0 years old). Educational backgrounds were as follows: high school: 4685 (31.8%), college/university: 9143 (62.0%), graduate school: 633 (4.3%), and others 280 (1.9%). Healthcare professionals were as follows: doctors: 159 (1.1%), pharmacists: 161 (1.1%), dieticians: 311 (2.1%).

### 3.2. Well-Balanced Diet

First, we asked participants regarding the frequency of consuming a well-balanced diet; 33.2% of them answered “almost every day” and 20.4% of them answered “4–5 days a week”, while 25.5% of them answered “rarely” (Table 1). Then, we further asked the participants who answered “rarely” why they did not have a well-balanced diet; 41.6% of the participants answered “I have no time to spare” and 36.9% of them answered “I do not have enough money.”

### 3.3. The Prevalence of Vitamin-Fortified Food and Vitamin Supplement Usage

The prevalence of vitamin-fortified food usage is shown in Table 2. We asked the participants their reason for answering “I do not care/I never used it” and the main reason was “I feel that I can get enough with my usual diet” (30.3%), followed by “I do not want to spend money on fortified foods” (28.5%), and “It is expensive to buy” (26.0%). The prevalence of vitamin-fortified food usage was associated with awareness of usual diets. Participants who usually consumed a well-balanced diet presented a greater prevalence of vitamin-fortified food usage than participants who did not consume a well-balanced diet (Table 3).

On the other hand, the prevalence of vitamin supplement usage was shown in Table 4. Then, we asked the participants their reason for answering “I have never used it”. The main reason was “I do not want to spend money on supplements” (38.3%), followed by “It is expensive to buy” (28.2%), and “I feel that I can get enough with my usual diet” (27.0%). Similar to vitamin-fortified food, the prevalence of vitamin supplement usage was also associated with awareness of usual diets. Participants who usually consumed a well-balanced diet presented a greater prevalence of vitamin supplement usage than participants who did not consume a well-balanced diet (Table 5).

Users of both fortified foods (“I actively use it” and “I use it occasionally”) and supplements (“I currently use it”) were 15.2%.

### 3.4. The Perception of Vitamins

When asked about the perception of each vitamin in four degrees, from “I understand its role in the body well” to “I do not know”, the vitamins for which the respondents answered with “I understand its role in the body well” were the highest in vitamin C (20.1%), then vitamin D (14.0%), and vitamin E (12.5%), and the lowest in pantothenic acid (5.6%) and biotin (5.6%) (Table 6). Perceptions ranged from “I understand its role in the body well” to “I have only heard about it”. In this regard, the perception was the highest in vitamin C (93.2%), then vitamin A (90.2%), and vitamin B_1_ (88.7%), and the lowest in biotin (41.9%).

### 3.5. Awareness of One’s Vitamin Intake

When asked about the awareness of their vitamin intake status in three degrees, from “Enough” to “I do not know”, the vitamins ranked by the proportions of answers of “Enough” were vitamin C (22.3%) in first place, followed by vitamin A (14.2%) and vitamin B_1_ (13.9%), and biotin was last (5.2%) (Table 7). The pattern was similar for the answer of “Insufficient”: vitamin C (30.0%) ranked first, and biotin (23.1%) was last. However, more than half of the participants answered “I do not know” for all the vitamins except for vitamin C (47.7%).

### 3.6. Conscious Intake of Vitamins

One-third of participants consciously took vitamin C (33.6%) (Table 8). The other vitamins were taken by almost 10%, and niacin, pantothenic acid, and biotin represented less than 2%. More than half of the participants took no vitamins consciously. There was a gender difference in conscious vitamin intake, and females were more conscious than males in almost all vitamins except for pantothenic acid.

### 3.7. Purpose of Use of Vitamin-Fortified Foods and Vitamin Supplements

We asked about the reasons for vitamin-fortified food/vitamin supplement use in these product users (*n* = 2000; 1000 males and 1000 females). The primary purpose of the use of these products was “Maintenance of health” (80.5%), followed by “Supplementation of nutrients” (47.8%), “Beauty benefits” (27.5%), and “Prevention of diseases” (27.2%) (Table 9). There were gender differences in the purpose of using these products: “Beauty benefits” was higher in females compared to males; on the other hand, “Building muscle” was higher in males compared to females.

### 3.8. Utilization of the Dietary Reference Intakes for Japanese People, Food Labeling on Products, and Application Programs

Among vitamin-fortified food/vitamin supplement users (2000 participants), only 8.3% of the participants used the DRIs-J (Table 10). Most of the participants had only heard of or did not know about the DRIs-J.

Vitamin contents are labeled on vitamin-fortified food/vitamin supplements. Labeling is the primary tool by which to recognize the amount of nutrients in a product. However, only 28.2% of surveyed vitamin-fortified food/vitamin supplement users always check it, and 52.9% of them sometimes check it (Table 11). Surprisingly, 2.3% of the participants did not know that vitamin contents were labeled on product packages, even though they used vitamin-fortified food/vitamin supplements. There were slight gender differences in the confirmation of labeling. According to the checking of nutrition labels, only 14.0% of the participants recognized the amount of each vitamin they were taking; 61.1% did not recognize it and 24.9% did not care about it.

There are many application programs (apps) for smartphones that can analyze nutrient contents in dishes upon taking pictures and scanning. It is helpful to recognize the amount of nutrients, including vitamins, that we get from foods. However, only 6.5% of the participants use these apps; 10.1% of the participants have used them in the past and 83.4% have never used them (Table 12).

## 4. Discussion

In this study, the perception and the prevalence of each vitamin showed varying results among all the vitamins considered. In addition, consumers were not aware of the nutritional status of each vitamin themselves, including vitamin-fortified food/vitamin supplement users. In Japan, the labeling of vitamins on product packages is voluntary. For this reason, most food products do not label vitamin contents on the package. However, labeling vitamin contents on packages of vitamin-fortified food/vitamin supplements is important information to know the amount of vitamin intake. We can also grasp usual vitamin intakes from food by the NHNS-J. This information helps to estimate the amounts of vitamins in these products. However, our study showed that most of the consumers were not aware of their levels of vitamin intake.

Within the framework of the Codex Alimentarius, guidelines for the use of nutrition and health claims at the international level have been established. In particular, the Codex Committee on Nutrition and Foods for Special Dietary Uses (CCNFSDU) reviews the general standards of food nutrition and nutritional reference values (NRVs), which are a set of values used in nutrition labeling derived from authoritative recommendations for daily nutrient intake, including that of vitamins and minerals [17]. These recommendations are based on the best available scientific knowledge of the daily amount of energy or nutrients needed for good health. NRVs usually do not appear on the label, but they are used in nutrition labeling to show the contribution of the nutrients to healthy nutrient intake in a portion of the food. Both the Japanese government and other countries follow the Codex guidelines on nutrient labeling systems.

Labeling is of importance to increase consumer awareness regarding food and products. A review article has been published concerning consumers’ perceptions and the usage of food labeling in Europe [18]. Some of the reports indicated that most consumers always or occasionally check nutritional information. However, it was also reported that these perceptions were estimated by self-reports and may have been overestimated. It is not clear to what extent consumers themselves can read and utilize these figures. Therefore, an easy-to-understand display is required, such as front-of-pack labeling (FOPL). CCNFSDU has started to work on the development of guidelines for FOPL. While international consistency is important, it seems important to have flexibility because the situation is different in each country. Since Japan has one of the highest salt intakes in the world, it is important to reduce salt intake [19,20]. On the other hand, Japan is the most aged society in the world, and prevention of sarcopenia/frailty is an important issue. In this regard, protein intake is encouraged. The efficacy of information, including FOPL, depends on the consistency of the target population and its message. However, there are few studies on whether nutrition labels are used in purchase decisions [18]. Recently, the efficacy of FOPL was reviewed, and a meta-analysis was conducted [21]. In this review, FOPL for sugar, calories, saturated fat, and sodium encouraged healthier food purchasing, but there are still few studies about the efficacy of FOPL for consumption. In addition, FOPL also encourages eliciting changes in food production and supply, including product reformulation, especially for unhealthy foods [22,23]. Thus, FOPL could be used to promote vitamin intake.

Inadequate food intake causes some problems. In Japan, the energy imbalance gap (average daily difference between energy intake and expenditure) was +2.3 kcal/day in males; on the other hand, it was −0.5 kcal/day in females in 2015. This gap was greater in obese men and underweight or normal weight women [24]. This trend causes obese men to become more obese and thin women to become thinner. In addition, it was reported that nutritional condition was also associated with infection and aggravation of acute upper respiratory infections, which included not only cold and influenza [25], but also SARS-CoV-2 [26]. Among vitamins, the role of vitamin D in COVID-19 is well researched, because vitamin D plays a role in the maintenance of the immune system and upper respiratory infections [27]. Lower plasma vitamin D levels are associated with the risk of COVID-19 infection and aggravation [28,29] and supplementation with vitamin D in COVID-19 patients was significantly associated with reduced ICU admission/mortality [30].

In addition to vitamin D, information on websites suggesting that various nutrients and food intakes are beneficial for preventing COVID-19 infection and aggravation is spreading in Japan and also around the world. Government authorities in each country and WHO have cautioned consumers about fake information and products that claim efficacy for the prevention of COVID-19 without any scientific evidence [31,32,33]. In addition, the WHO sent a message for COVID-19, titled “Nutrition advice for adults during the COVID-19 outbreak” [34]. It includes five messages as follows: “Eat fresh and unprocessed foods every day.”, “Drink enough water every day.”, “Eat moderate amounts of fat and oil.”, “Eat less salt and sugar.”, and “Avoid eating out.”. The contents introduced here are similar to those of the “healthy diet” originally issued by the WHO [35]. In other words, the usual healthy diets are important in the prevention of infectious diseases, including COVID-19. In Japan, the use of the Japanese Food Guide Spinning Top is recommended for a well-balanced diet [36], and it is reported that awareness of the Japanese Food Guide Spinning Top was associated with BMI and waist circumstance via eating behavior [37]. However, it is difficult to always keep in mind a well-balanced diet, and, in particular, it may be difficult to grasp the intake of micronutrients (vitamins and minerals), so it is necessary to keep in mind the intake. Moreover, anemia and osteoporosis are important issues in females. The risk of these diseases is associated with iron and calcium intake, respectively. In this regard, nutrition education and environments, such as canteens [38] or school lunch programs [39] for children, are important to encourage not only children but also their parents to care about food intake. We are planning to research the perception and prevalence of each mineral and its association with education levels in our next study.

Currently, the Ministry of Health, Labour and Welfare is discussing how to establish sustainable environments in which consumers can automatically become healthy. In this committee, some manufactures have introduced their engagements. Even though they have modified the products to be better for health compared to the previous ones, consumers did not choose them if they were more expensive compared to previous ones. Our results showed that many participants did not use vitamin-fortified food/vitamin supplements because of the price, as indicated in their comments. In this regard, consumers need products that are nutrient-rich without increased prices. Moreover, it is ideal to be able to get enough nutrients from any diet combinations without any considerations.

The strength of this study is that this is the first report that clarified the perception of each vitamin and their prevalence in fortified foods and supplements. In addition, this survey was conducted on 14,741 participants. On the other hand, a limitation is that this study was an online survey, so the participants were registrants of the survey company. In this regard, we have to carefully treat our data as general, even though internet and online questionnaires are popular across all age groups.

## 5. Conclusions

In this study, 33.2% of the participants consumed a well-balanced diet every day. The prevalence of vitamin-fortified food usage and vitamin supplement usage was 10.0% and 21.4%, respectively, and these were higher in participants who usually consumed a well-balanced diet. The purposes for the usage of these products were varied, such as maintaining health (80.5%) or supplementation of nutrients (47.8%). Most participants did not use vitamin-fortified food and/or vitamin supplements due to economic reasons. To remedy nutritional status in individuals, it is important to improve not only consumer awareness but also the environment, which can lead consumers to an appropriate product selection without any economic burden.

## Figures and Tables

**Table 1:** Frequency of a well-balanced diet and reasons for its lack.

	*n*	%
Almost every day	4900	33.2
4–5 days a week	3007	20.4
2–3 days a week	3078	20.9
Rarely	3756	25.5
Reasons for “Rarely” (*n* = 3756)		
I have no time to spare	1562	41.6
I do not have enough money	1386	36.9
There are many opportunities to eat out	413	11.0
I often buy lunch boxes and prepared dishes	1192	31.7
Others	376	10.0

**Table 2:** The prevalence of vitamin-fortified food usage and reasons for non-usage.

	*n*	%
I actively use it.	1477	10.0
I use it occasionally.	3332	22.6
I do not care./I have never used it.	9932	67.4
Reasons for not using (*n* = 9932)		
I feel that I can get enough with my usual diet.	3014	30.3
No vitamin-fortified foods that I want to consume.	784	7.9
I use other products such as supplements.	929	9.4
It is expensive to buy.	2586	26.0
I do not want to spend money on fortified foods.	2832	28.5
I have bought it, but it was not delicious.	278	2.8
I have bought it, but it did not suit my health.	766	7.7
Others	589	5.9

**Table 3:** The prevalence of vitamin-fortified food usage and reasons for non-usage.

	*n*	I Currently Use It.	I Used to Use It, but Not Now.	I Do Not Care./ I Have Never Used It.	*p*-Value
Almost every day	4900	16.2	22.6	61.3	<0.001
4–5 days a week	3007	10.8	30.4	58.8	
2–3 days a week	3078	7.0	26.6	66.4	
Rarely	3756	3.9	13.1	83.0	

The difference among groups was examined using the Chi-square (χ^2^) test.

**Table 4:** The prevalence of vitamin supplement usage and reasons for non-usage.

	*n*	%
I currently use it.	3160	21.4
I used to use it, but not now.	3178	21.6
I have never used it.	8403	57.0
Reasons for not using (*n* = 8403)		
I feel that I can get enough with my usual diet.	3129	27.0
No vitamin supplements that I want to consume.	1002	8.7
I use other products such as fortified foods.	483	4.2
It is expensive to buy.	3271	28.2
I do not want to spend money on supplements.	4430	38.3
I have bought it but it did not suit my health.	1084	9.4
Others	659	5.7

**Table 5:** The relationship between frequency of a well-balanced diet and prevalence of vitamin supplement usage.

	*n*	I Currently Use It.	I Used to Use It, but Not Now.	I Have Never Used It.	*p*-Value
Almost every day	4900	25.7	21.2	53.1	<0.001
4–5 days a week	3007	25.5	26.3	48.1	
2–3 days a week	3078	20.3	25.2	54.5	
Rarely	3756	13.4	15.2	71.3	

The difference among groups was examined using the chi-square (χ^2^) test.

**Table 6:** The perception of each vitamin.

	I Understand Its Role in the Body Well	I Understand Somewhat	I Have Only Heard about It	I Do Not Know	Perception ^1^	*p*-Value
Vitamin A	10.7	38.0	41.5	9.8	91.2	<0.001
Vitamin B_1_	9.3	36.3	43.1	11.3	88.7	
Vitamin B_2_	8.6	34.4	43.0	14.0	86.0	
Vitamin B_6_	7.4.	28.8	39.3	24.5	75.5	
Vitamin B_12_	7.7	26.7	36.3	29.3	70.7	
Vitamin C	20.1	43.4	29.7	6.8	93.2	
Vitamin D	14.0	36.5	36.9	12.6	87.4	
Vitamin E	12.5	34.4	37.6	15.5	84.5	
Vitamin K	7.5	24.4	33.9	34.1	65.9	
Niacin	6.2.	19.2	34.5	40.1	59.9	
Pantothenic acid	5.6	16.6	28.1	49.8	50.2	
Folate	9.6	27.5	42.9	20.1	79.9	
Biotin	5.6	13.9	22.4	58.1	41.9	

^1^ Perception ranges from “I understand its role in the body well” to “I have only heard about it”. The difference among vitamins was examined using the chi-square (χ^2^) test.

**Table 7:** The perception of one’s vitamin intake status.

	Enough	Insufficient	I Do Not Know	*p*-Value
Vitamin A	14.2	27.8	58.0	<0.001
Vitamin B_1_	13.9	27.5	58.6	
Vitamin B_2_	12.9	27.0	60.1	
Vitamin B_6_	10.8	26.8	62.4	
Vitamin B_12_	10.4	26.6	63.0	
Vitamin C	22.3	30.0	47.7	
Vitamin D	13.2	28.9	57.9	
Vitamin E	11.7	27.7	60.6	
Vitamin K	7.7	26.0	66.2	
Niacin	6.1	24.2	69.7	
Pantothenic acid	5.7	23.8	70.5	
Folate	7.9	26.6	65.5	
Biotin	5.2	23.1	71.7	

The difference among vitamins was examined using the chi-square (χ^2^) test.

**Table 8:** Consciously ingested vitamins.

	All (%)	Male	Female	*p*-Value
Vitamin A	10.1	9.3	11.0	<0.001
Vitamin B_1_	10.7	10.0	11.3	
Vitamin B_2_	10.0	8.9	11.2	
Vitamin B_6_	7.5	7.0	8.0	
Vitamin B_12_	7.1	6.5	7.7	
Vitamin C	33.6	28.5	38.8	
Vitamin D	10.0	8.5	11.6	
Vitamin E	8.9	7.2	10.6	
Vitamin K	2.6	2.2	2.9	
Niacin	1.7	1.7	1.7	
Pantothenic acid	1.5	1.6	1.4	
Folate	5.8	3.1	8.6	
Biotin	1.6	1.5	1.8	
None	57.0	63.0	50.8	

The difference between males and females was examined using the chi-square (χ^2^) test.

**Table 9:** Purpose of use of vitamin-fortified food/vitamin supplements.

	All	Male	Female	*p*-Value
Maintenance of health	80.5	84.2	76.7	<0.001
Supplementation of nutrients	47.8	49.1	46.4	
Beauty benefits	27.5	11.1	43.9	
Weight loss	11.4	10.6	12.1	
Building muscle	9.7	13.3	6.1	
Improvements to health	18.6	18.3	18.9	
Prevention of diseases	27.2	30.0	24.3	
Treatment of diseases	6.3	6.1	6.5	
Improvement of immune function/ prevention of infectious diseases	23.2	22.3	24.0	
Other	1.4	0.6	2.1	

*n* = 2000. The difference between males and females was examined using the chi-squared (χ^2^) test.

**Table 10:** Perception and utilization of the dietary reference intakes for Japanese people.

	All	Male	Female	*p*-Value
I know about it and I use it.	8.3	8.9	7.7	0.072
I know about it, but I do not use it.	15.4	14.8	15.9	
I have only heard about it.	42.9	40.5	45.2	
I do not know about it.	33.5	35.8	31.2	

*n* = 2000. The difference between males and females was examined using the chi-squared (χ^2^) test.

**Table 11:** Confirmation of labeling of vitamin contents on fortified food/supplements.

	All	Male	Female	*p*-Value
I always check it.	28.2	30.8	25.5	0.025
I sometimes check it.	52.9	49.9	55.8	
I do not check it.	16.8	16.7	16.8	
I did not know that it was labeled.	2.3	2.6	1.9	

*n* = 2000. The difference between males and females was examined using the chi-squared (χ^2^) test.

**Table 12:** Utilization of application programs for nutrition analysis in foods.

	All	Male	Female	*p*-Value
I currently use them.	6.5	8.4	4.6	<0.001
I used to use them, but not now.	10.1	11.5	8.7	
I have never used them.	83.4	80.1	86.7	

*n* = 2000. The difference between males and females was examined using the Chi-squared (χ^2^) test.

## Data Availability

The data presented in this study are available on request from the corresponding author.

## Supplementary Materials

The following are available online at https://www.mdpi.com/article/10.3390/nu13093136/s1, Supplementary Materials—Questionnaire.
