# Supplementary material for: The Perception of Vitamins and Their Prevalence in Fortified Food and Supplements in Japan
Source: Nutrients. 2021 Sep 8;13(9):3136. doi: 10.3390/nu13093136 (PMC8469082; doi:10.3390/nu13093136)
Supplement: Supplementary file 1 [file nutrients-13-03136-s001.zip › nutrients-1359837-supplementary.pdf]

Questionnaire

Q1-1. How often do you consume a well-balanced diet?

A well-balanced diet is having a meal consisting of the following three dishes at least twice a day.
Grain dish (rice, bread, noodles, etc.)
Main dish (dishes mainly made of meat, fish, eggs, soybeans, soy products, etc.)
Side dish (dishes mainly made of vegetables, seaweed, mushrooms, etc.)

1. Almost every day
2. 4-5 days a week
3. 2-3 days a week
4. Rarely

Q1-2. What is(are) the reason(s) you cannot consume a well-balanced diet? (Multiple answers)

1. I have no time to spare.
2. I do not have enough money.
3. There are many opportunities to eat out.
4. I often buy lunch boxes and prepared dishes.
5. Others

Q2-1. Do you use vitamin-fortified food?

"Vitamin-fortified food" are foods that are sold as a regular food, but are labeled as "added vitamin X xx mg", "vitamin X xx% fortified", etc.

1. I actively use it.
2. I use it occasionally.
3. I do not care. / I have never used it.

Q2-2. What is(are) the reason(s) for not using vitamin-fortified food? (Multiple answers)

1. I feel that I can take enough with my usual diet.
2. No vitamin-fortified foods that I want to consume.
3. I use other products such as supplements.
4. It is expensive to buy.
5. I do not want to spend money on fortified foods.
6. I have bought it, but it was not delicious.
7. I have bought it, but it did not suit my health.
8. Others

Q3-1. Do you use vitamin supplement?

There is no definition of supplement in Japan. In this survey, capsules, tablets, and other forms that containing condensed specific ingredients are defined as supplement.

1. I currently use it.
2. I used to use it, but not now.
3. I have never used it.

Q3-2. What is(are) the reason(s) for not using vitamin supplement? (Multiple answers)

1. I feel that I can take enough with my usual diet.
2. No vitamin supplements that I want to consume.
3. I use other products such as fortified foods.
4. It is expensive to buy.
5. I do not want to spend money on supplements.
6. I have bought it but it did not suit my health.
7. Others

Q4. How much do you know about each vitamin?

	I understand its role in the body well.	I understand somewhat.	I have only heard about it.	I do not know.
Vitamin A				
Vitamin B_1				
Vitamin B_2				
Vitamin B_6				
Vitamin B_{12}				
Vitamin C				
Vitamin D				
Vitamin E				
Vitamin K				
Niacin				
Pantothenic acid				
Folate				
Biotin				

Q5. How do you think of your vitamin intake status?

	Enough	Insufficient	I do not know
Vitamin A			
Vitamin B_1			
Vitamin B_2			
Vitamin B_6			
Vitamin B_{12}			
Vitamin C			
Vitamin D			
Vitamin E			
Vitamin K			
Niacin			
Pantothenic acid			
Folate			
Biotin			

Q6. Choose the vitamins that you consciously ingest.

Vitamin A	
Vitamin B_1	
Vitamin B_2	
Vitamin B_6	
Vitamin B_{12}	
Vitamin C	
Vitamin D	
Vitamin E	
Vitamin K	
Niacin	
Pantothenic acid	
Folate	
Biotin	
None	

(Following questions are asked to only vitamin-fortified food/vitamin supplement users.)

Q7. What is(are) the purpose(s) to use vitamin-fortified food/vitamin supplement? (Multiple answers)

1. Maintenance of health
2. Supplementation of nutrients
3. Beauty benefits
4. Weight loss
5. Building muscle
6. Improvements to health
7. Prevention of diseases
8. Treatment of diseases
9. Improvement of immune function / prevention of infectious diseases
10. Other

Q8. Do you know "the Dietary Reference Intakes for Japanese"?

"The Dietary Reference Intakes for Japanese" proposes reference values for the intake of energy and nutrient to promote and maintain heath, and to prevent lifestyle-related diseases.

1. I know about it and I use it.
2. I know about it, but I do not use it.
3. I have only heard about it.
4. I do not know about it.

Q9. Do you check vitamin content labeled on fortified food/supplement packages?

1. I always check it.
2. I sometimes check it.
3. I do not check it.
4. I did not know that it was labeled.

Q10. Do you use application programs that tells calories and nutrient contents by taking a photo of your meal?

1. I currently use them.
2. I used to use them, but not now.
3. I have never used them.
